# Supplementary material for: Divergent Mechanisms of H2AZ.1 and H2AZ.2 in PRC1-Mediated H2A Ubiquitination
Source: Cells. 2025 Jul 23;14(15):1133. doi: 10.3390/cells14151133 (PMC12346162; doi:10.3390/cells14151133)
Supplement: Supplementary file 1 [file cells-14-01133-s001.zip › cells-3727363-Supplementary-Figure_S1-S7_06122025.pdf]

Figure S1.

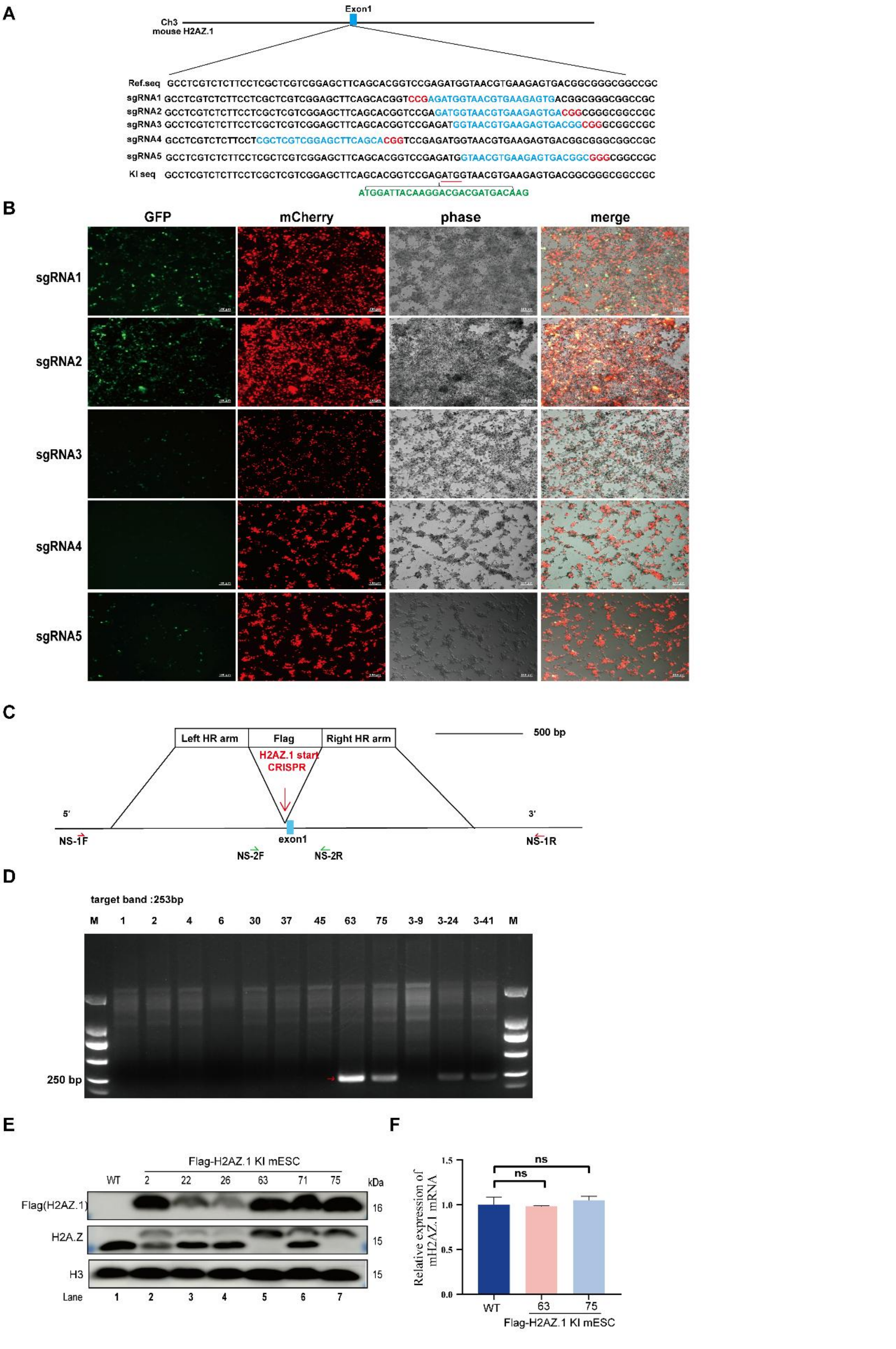

Figure S1. Strategy for generating Flag-H2AZ.1 knockin mouse ESC lines.

A. Schematic of gRNA design targeting H2AZ.1 gene regions near ATG, with sgRNA

sequences in blue, PAM sequences in red, and ATG underlined.

B. GFP/mCherry assays assessing the efficiency of H2AZ.1 sgRNAs in inducing double-strand breaks.

C. Schematic for inserting a Flag tag into the H2AZ.1 gene using a rescue template with 1000 bp homologous arms. Primers for identifying positive colonies are shown below, and sequences of correctly targeted Flag-H2AZ.1 KI ESC clones are indicated in panel A (green).

D. Agarose gel imaging of PCR products from individual clones of Flag-H2AZ.1 knock-in (KI) mESCs. Correctly targeted bands are indicated with red arrows.

E. Immunoblots of control (WT R1 ESC) and candidate Flag-H2AZ.1KI mouse ESC colonies.

F. RT-qPCR analysis of *H2AZ.1* transcript levels normalized to GAPDH in WT (wild type) and H2AZ.1 monoclonal cells.

Figure S2

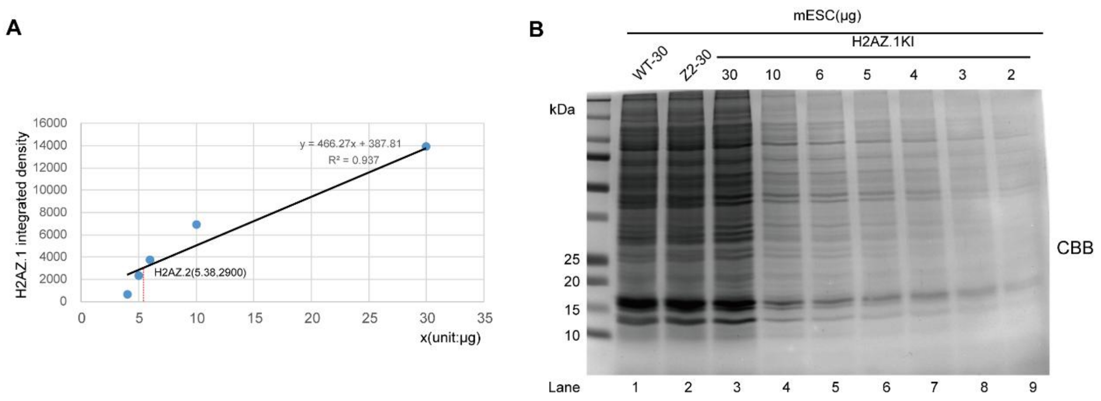

Figure S2. Quantification of the H2AZ.2 protein levels relative to H2AZ.1.

A. Quantification of H2AZ.2 protein levels by titration of the amount of H2AZ.1.

B. Coomassie Brilliant Blue (CBB)-stained SDS-PAGE, showing protein profiles in Figure 1C.

Figure S3.

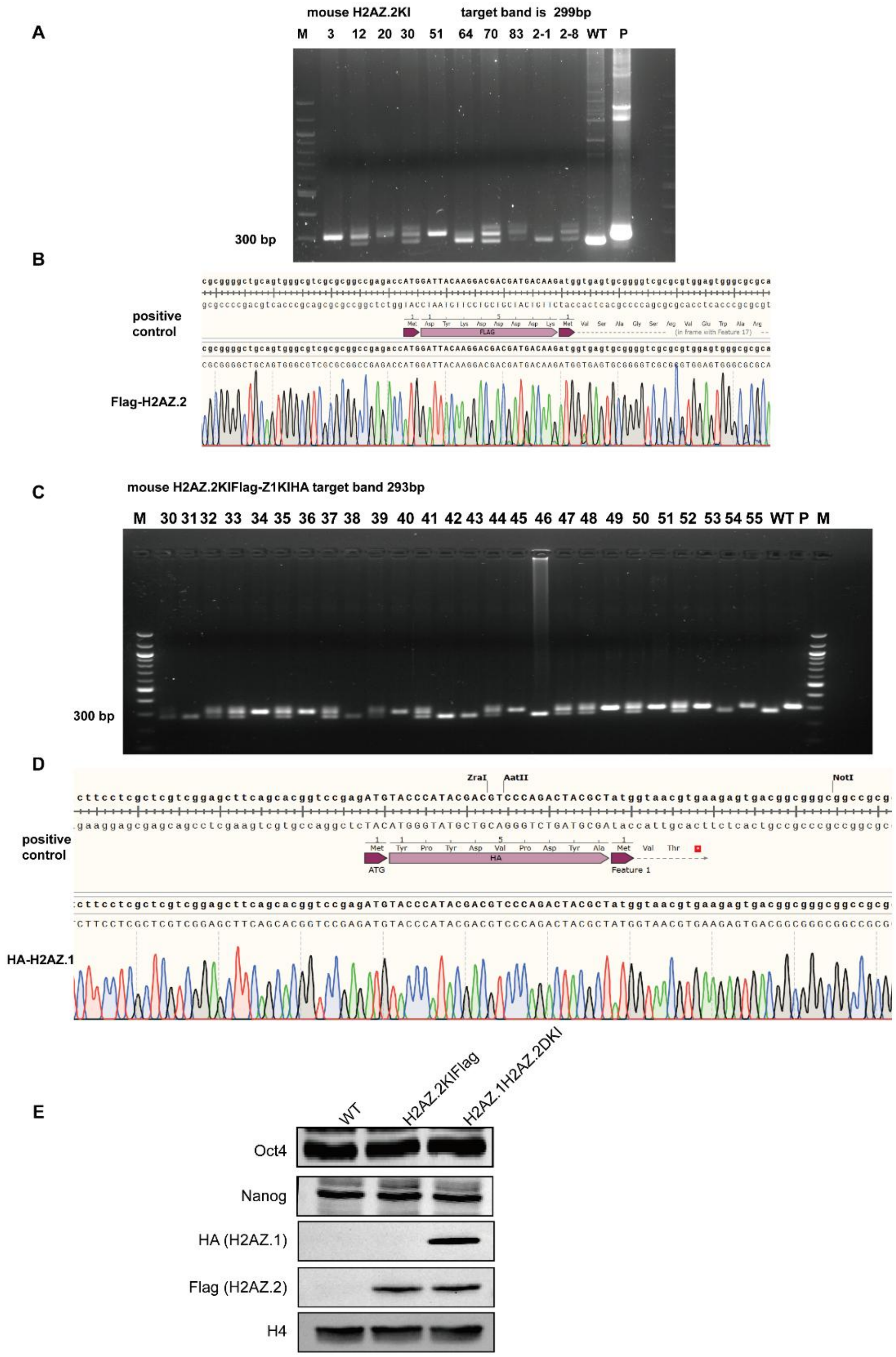

Figure S3. Strategy for generating HA-H2AZ.1 and Flag-H2AZ.2 double KI mESC lines.

- **A.** Agarose gel imaging of PCR products from wild-type (WT) mESC R1 (NC, negative control), rescue template (P, positive control), and individual colonies from Flag-H2AZ.2 KI experiments.
- **B.** Sanger sequencing of the PCR products from one H2AZ.2KI mESC clone confirmed the correct insertion of Flag tag into the *H2afv* gene (encoding H2AZ.2) with no mutations.
- **C.** Agarose gel imaging of PCR products from Flag-H2AZ.2 KI mESCs (WT), correctly

targeted template (P), and individual colonies from knocking HA-H2AZ.1 in Flag-H2AZ.2 mESCs.

D. Sanger sequencing of the H2AZ.1 knock-in allele confirms accurate insertion of the HA tag with no mutations.

E. Immunoblots of control (mESC R1), Flag-H2AZ.2 KI mESC lines, and HA-H2AZ.1/Flag-H2AZ.2 DKI mESCs. H4 was used as the loading control.

Figure S4

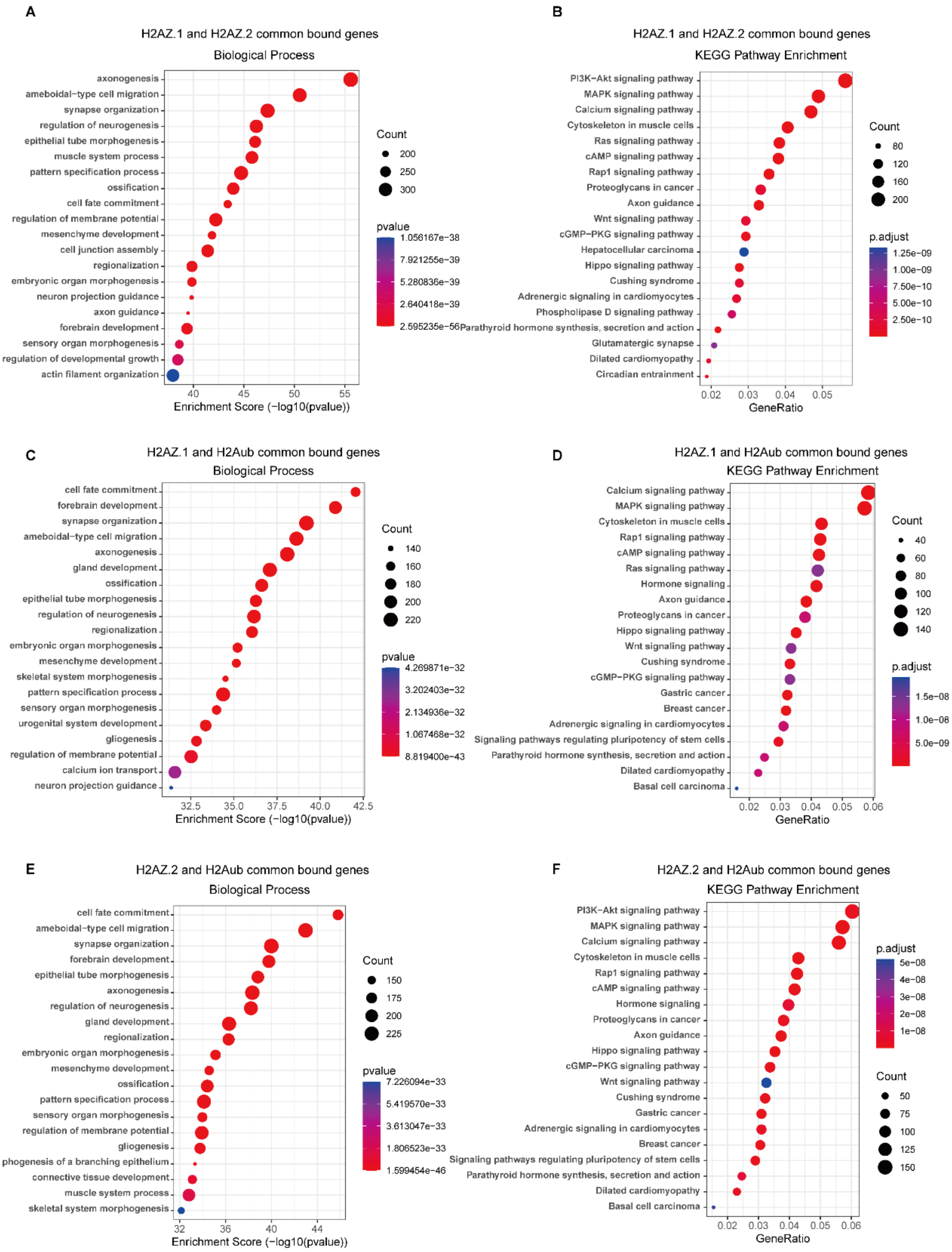

Figure S4. Gene Ontology (GO) and KEGG pathway enrichment analyses of genes bound by H2AZ isoforms and H2AK119ub.

A–B. GO biological process (A) and KEGG pathway (B) enrichment analyses of genes commonly bound by H2AZ.1 and H2AZ.2. Enriched terms include developmental processes such as axonogenesis, neurogenesis, and cell fate commitment, as well as key signaling pathways including PI3K-Akt, MAPK, and calcium signaling.

C–D. GO biological process (C) and KEGG pathway (D) enrichment analyses of genes commonly bound by H2AZ.1 and H2Aub. The shared targets are associated with differentiation-related processes (e.g., mesenchyme development, synapse organization) and signaling pathways (e.g., Hippo, Wnt, Ras, Rap1).

E–F. GO biological process (E) and KEGG pathway (F) enrichment analyses of genes commonly bound by H2AZ.2 and H2Aub. These genes are enriched in similar biological processes and pathways related to development, morphogenesis, and stem cell regulation. Dot size indicates the number of genes associated with each term or pathway, and color gradient reflects statistical significance (adjusted p-values).

Figure S5

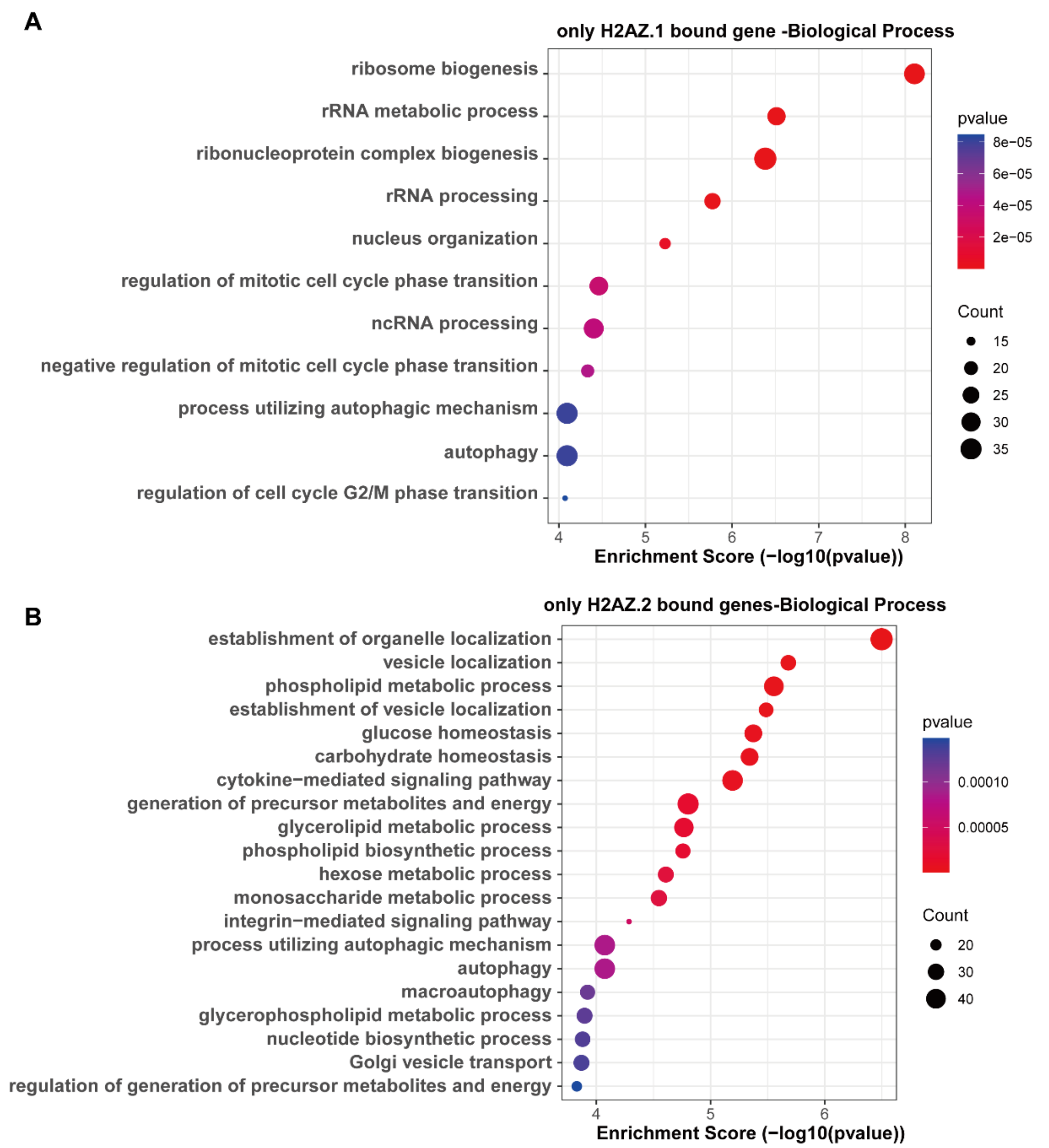

Figure S5 Distinct Biological Processes Enriched in Genes Specifically Bound by H2AZ.1 and H2AZ.2

A–B. GO biological process enrichment analyses of genes specifically bound by H2AZ.1 (A) and H2AZ.2 (B). Dot size indicates the number of genes associated with each GO term, and color gradient reflects statistical significance (p value), with red indicating more significant enrichment.

Figure S6

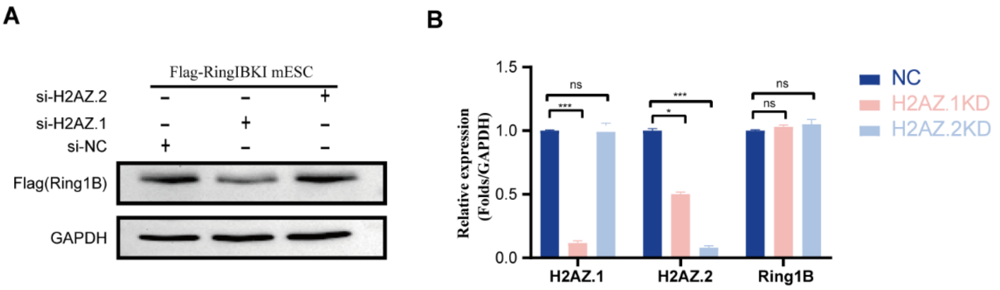

Figure S6. Validation of H2AZ.1 and H2AZ.2 knockdown in CUT&Tag samples.

A. Western blot analysis of Flag-Ring1B expression in Ring1B KI mouse embryonic stem cells (mESCs) following siRNA-mediated knockdown of H2AZ.1 or H2AZ.2. GAPDH was used as a loading control.

B. Quantitative RT-PCR analysis showing the relative mRNA expression levels of H2AZ.1, H2AZ.2, and Ring1B after knockdown. H2AZ.1KD and H2AZ.2KD effectively reduced the expression of their respective targets without significantly affecting Ring1B levels. Data are presented as mean ± SEM. ***$p < 0.001$; ns, not significant.

Figure S7

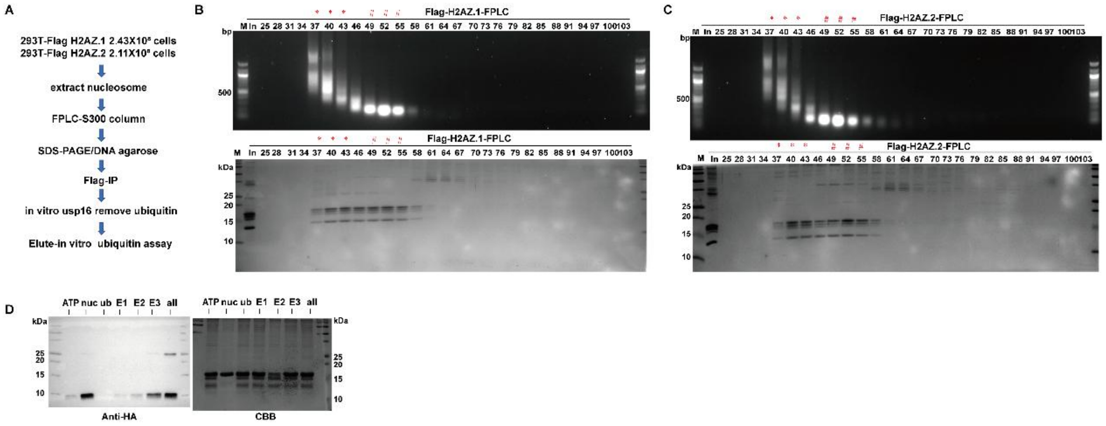

Figure S7. Purification of Flag-H2AZ.1 and Flag-H2AZ.2 nucleosomes.

A. Schematic workflow of the experimental procedure. The workflow includes: nucleosome extraction from Flag-H2AZ.1 and H2AZ.2 293T cells, fractionation of nucleosomes into mono- and oligo- nucleosomes using Sephacryl S-300, immunoprecipitation with Flag antibody to purify the H2AZ.1 and H2AZ.2-containing nucleosomes, USP16 treatment to remove H2Aub on these nucleosomes, and PRC1-mediated in vitro ubiquitin ligase assays.

B. SYBR-stained DNA agarose gel (top) and a CBB-stained SDS-PAGE (bottom) showing profiles of Sephacryl S300 column fractions for H2AZ.1. DNA from the indicated fractions was extracted and analyzed using 1.5% agarose gel

electrophoresis. An aliquot of S300 column fractions was separated on an 18% SDS-PAGE. M represents the molecular weight marker, and In corresponds to the input sample before fractionation.

C. SYBR-stained DNA agarose gel (top) and a CBB-stained SDS-PAGE (bottom) showing profiles of Sephacryl S300 column fractions for H2AZ.2. DNA from the indicated fractions was extracted and analyzed using 1.5% agarose gel electrophoresis. An aliquot of S300 column fractions was separated on an 18% SDS-PAGE. M represents the molecular weight marker, and In corresponds to the input sample before fractionation.

D. In vitro ubiquitination assay using HA-tagged ubiquitin. Reaction components include ATP, nucleosome (nuc), ubiquitin (ub), E1, E2, E3, and a complete reaction mix (all). Samples were analyzed by SDS-PAGE followed by immunoblotting with an anti-HA antibody (left panel) to detect ubiquitinated proteins. CBB stained (right panel) was shown. Molecular weight markers (in kDa) are shown on the left.
