# Supplementary material for: Divergent Mechanisms of H2AZ.1 and H2AZ.2 in PRC1-Mediated H2A Ubiquitination
Source: Cells. 2025 Jul 23;14(15):1133. doi: 10.3390/cells14151133 (PMC12346162; doi:10.3390/cells14151133)
Supplement: Supplementary file 1 [file cells-14-01133-s001.zip › cells-3727363-Supplementary_Table_S1_07072025.pdf]

siRNA constructs	Sequence (5'-3')
mouseRing1B-sense-1	rUrArUrUrUrCrUrCrArCrUrGrArCrCrArArUrUrCrCrArArArGrArA
mouseRing1B-anti sense-1	rCrUrUrUrGrGrArArUrUrGrGrUrCrArGrUrGrArGrArArATA
mouseRing1B-sense-2	rArUrGrCrArArArCrArUrGrUrCrUrUrUrGrUrArArArUrArCrUrGrU
mouseRing1B-anti sense-2	rArGrUrArUrUrUrArCrArArArGrArCrArUrGrUrUrUrGrCAT
mouseH2AZ.1-sense-1	rArGrGrUrCrArUrArUrCrCrCrArArArCrUrArGrCrUrUrUAA
mouseH2AZ.1-anti sense-1	rUrUrArArArGrCrUrArGrUrUrUrGrGrGrArUrArUrGrArCrCrUrUrU
mouseH2AZ.1-sense-2	rGrCrUrUrArArUrArArUrCrGrGrGrArCrUrUrArCrCrUrUAG
mouseH2AZ.1-anti sense-2	rCrUrArArGrGrUrArArGrUrCrCrCrGrArUrUrArUrUrArArGrCrArA
mH2AZ.2-1-sense	rArArArArArUrGrUrUrUrUrArUrCrArArUrUrCrUrArCrUrUrUrUrG
mH2AZ.2-1-Anti-Sense	rArArArGrUrArGrArArUrUrGrArUrArArArArCrArUrUrUTT
mH2AZ.2-2-sense	rCrCrUrUrCrUrUrUrCrCrArArUrCrArGrArGrArCrUrUrGrUrGrGrA
mH2AZ.2-2-Anti-Sense	rCrArCrArArGrUrCrUrCrUrGrArUrUrGrGrArArArGrArAGG
mouse negative control-sense	rCrGrUrUrArArUrCrGrCrGrUrArUrArArUrArCrGrCrGrUAT
mouse negative control-anti ser	rArUrArCrGrCrGrUrArUrUrArUrArCrGrCrGrArUrUrArArCrGrArC
mouse Ring 1A siRNA was purchased from Dharmacon（Ref:SO-3197417G）	

primer for gene editing	Sequence (5'-3')
mH2AZ.2KI-1spacerF	CACC GCGCGACCCCGCACTCACCAGTTTC
mH2AZ.2KI-1spacerR	CTCTGAAAC TGGTGAGTGCGGGGTCGCGC
mH2AZ.2KI-2spacerF	CACC GCGTCGCGCGGCCGAGACCAGTTTC
mH2AZ.2KI-2spacerR	CTCTGAAAC TGGTCTCGGCCGCGCGACGC
mH2AZ.2KI-1PR-F	GTGCGCGCGGGGCTGCATGGGCGTCGCGCGGCCGAGACCATGGATTACAAGGACGACGATGACAAGATGGTGAGTGCGGGG
mH2AZ.2KI-1PR-R	CGCGCCCCGCACTCACCATCTTGTCATCGTCGTCCTTGTAATCCATGGTCTCGGCCGCGCGACGCCCACTGCAGCCCCGCGC
mH2AZ.2KI-2PR-F	GTGCGCGCCCACTCCACGCGCGACCCCGCACTCACCATCTTGTCATCGTCGTCCTTGTAATCCATGGTCTCGGCCGCGCGAC
mH2AZ.2KI-2PR-R	CGCGGTCGCGCGGCCGAGACCATGGATTACAAGGACGACGATGACAAGATGGTGAGTGCGGGGTCGCGCGTGGAGTGGGCGC
mH2AZ.1KI-12spacerF	CACCGACTCTTCACGTTACCATCTGTTTC
mH2AZ.1KI-12spacerR	CTCTGAAACAGATGGTAACGTGAAGAGTC
mH2AZ.1KI-1PR-F	GTGCTTCAGCACGGTCCGAGATGTACCCATACGACGTCCCAGACTACGCTATGGTAACGTGAAGAG
mH2AZ.1KI-1PR-R	CGCGCTCTTCACGTTACCATAGCGTAGTCTGGGACGTCGTATGGGTACATCTCGGACCGTGCTGAA
mH2AZ.1KI-2PR-F	GTGCAGCTTCAGCACGGTCCGAGATGTACCCATACGACGTCCCAGACTACGCTATGGTAACGTGAAGAG
mH2AZ.1KI-2PR-R	CGCGCTCTTCACGTTACCATAGCGTAGTCTGGGACGTCGTATGGGTACATCTCGGACCGTGCTGAAGCT
mH2AZ.1-RT-F	ATCTAGGACAACCAGCCACG

mH2AZ.1-RT-R	TAGCAAGCTGCAAGTGACGA
mH2AZ.2-RT-F	CAATCCGCGGTGATGAAGAG
mH2AZ.2-RT-R	CAGAGACTTGTGGATGTGCG
mRing1B-RT-F	TTGCGCGGATTGTATTATCA
mRing1B-RT-R	CCTGATGCGCTTCATACTCA
mRing1A-RT-F	GAGGAATACGAGGCCCATCA
mRing1A-RT-R	CATTGTGGCGGTCTGATCAG
PE-mH2AZ.2-JD-F1	ACAAGGACGACGATGACAAG
PE-mH2AZ.2-JD-R1	AGTCCGGAACAGCGGGCACT
PE-mH2AZ.2-JD-F2	CCGAGGTTCAAACGAACAAC
PE-mH2AZ.2-JD-R2	AGTCCGGAACAGCGGGCACT
PE-mH2AZ.1KI-JD-F1	TATAAAGGGCGCGAGGAAGG
PE-mH2AZ.1KI-JD-F2	AGTTTGAATCGCGGTCCGAC
PE-mH2AZ.1KI-JD-R1	TAGTCTGGGACGTCGTATGG
PE-mH2AZ.1KI-JD-R2	TCCGGGGCTCCGAAAACACCT
mH2AZ.1-RT-F	ATCTAGGACAACCAGCCACG
mH2AZ.1-RT-R	TAGCAAGCTGCAAGTGACGA
mH2AZ.1KI-NS-1F	ACTGCTTGTGAATCATGTTA
mH2AZ.1KI-NS-1R	GGAGGGTGCAGACAAACAAG
mH2AZ.1KI-NS-2F	GTTCTCCCATTGGCTGGAG
mH2AZ.1KI-NS-2R	GTCCTTGTAATCCATCTCG
pegRNAscaffold-F	AGAGCTATGCTGGAAACAGCATAGCAAGTTGAAATAAGGCTAGTCCGTTATCAACTTGAAAAAGTGGCACCGAGTCG
pegRNAscaffold-R	GCACCGACTCGGTGCCACTTTTTCAAGTTGATAACGGACTAGCCTTATTTCAACTTTATGCTGTTTCCAGCATAG
